# Postload Plasma Glucose but Not Fasting Plasma Glucose Had a Greater Predictive Value for Cardiovascular Disease in a Large Prospective Cohort Study in Southwest China

**DOI:** 10.3389/fcvm.2021.815357

**Published:** 2022-01-24

**Authors:** Yingying Wang, Lisha Yu, Yiying Wang, Jie Zhou, Yanli Wu, Tao Liu, Na Wang, Chaowei Fu

**Affiliations:** ^1^Key Laboratory of Public Health Safety, NHC Key Laboratory of Health Technology Assessment, School of Public Health, Fudan University, Shanghai, China; ^2^Guizhou Province Center for Disease Prevention and Control, Chronic Disease Prevention and Cure Research Institute, Guiyang, China

**Keywords:** 2-h postload glucose, fasting plasma glucose, glycemia, cardiovascular disease, Southwest China, cohort study

## Abstract

**Background:**

Uncertainty remains regarding the relevance of glycemia, though below the threshold for diabetes, for the risk of cardiovascular disease (CVD) among the Southwest Chinese. We aimed to examine the associations of the first-onset CVD with fasting plasma glucose (FPG) and 2-h postload glucose (2h-PG) in Southwest China.

**Methods:**

The current study examined data from the Guizhou Population Health Cohort Study (GPHCS) of 9,280 participants aged 18 to 95 years recruited from 12 areas since 2010 in Guizhou Province, Southwest China. Participants were followed-up until December, 2020. Primary outcomes were the first onset of a composite of or one of major CVD events, including ischaemic stroke, haemorrhagic stroke and myocardial infarction. FPG, 2h-PG, other metabolic factors and some demographic factors were collected at baseline. Cox proportional hazards models were used to estimate the risk of CVD associated with FPG and 2h-PG. Sensitive analysis and stratified analysis were conducted among participants across different modifiable risk factors and demographic features

**Results:**

During a median of 6.58 years of follow-up, of 7,593 participants with available data for analysis, 174 experienced at least one CVD events, 158 developed stroke (including 126 ischemic stroke and 39 Ischemic stroke events), and 24 developed myocardial infarction. The risk of major CVD events was significantly increased with elevated 2h-PG but not FPG. Compared with participants in the lowest tertile of 2h-PG, those in the highest tertile had a 1.87-fold (95%CI: 1.26–2.77) increased risk for overall CVD, a 1.82-fold (95%CI: 1.20–2.75) increased risk for overall Stroke, and a 1.82-fold (95%CI: 1.20–2.75) increased risk for ischemic stroke, respectively, after adjustment for age, sex, smoking, ethnic group, education level, systolic blood pressure (SBP), triglycerides (TG), body mass index (BMI) and waist circumference (WC). However, there was no relation of glycemia of haemorrhagic stroke or myocardial infarction (*P* > 0.05). The effect sizes in the associations of CVD with 2h-PG become enhanced among those within normal range of glycemia, SBP, TG, BMI, as well as those without hypertension, dyslipidemia.and obesity.

**Conclusions:**

2h-PG, in contrast to FPG, is a significant indicator in predication of CVD in Southwest Chinese. Elevated 2h-PG, though below the below the threshold for diabetes, remains independently increased the risk of CVD.

## Introduction

Cardiovascular disease (CVD) is the leading cause of morbidity and mortality worldwide, with ischemic heart disease (IHD) and stroke as the main contributors (1). The burden of CVD in China has rapidly and substantially increased during the past two decades (2). IHD causes more than 1 million deaths per year, and the number of individuals with acute myocardial infarction (AMI) will increase to 23 million by 2030 (3). Unlike in western countries, the epidemic profile of stroke in China surpasses that of IHD, with annual estimates of 11 million prevalent cases, 2.4 million incident cases and 1.1 million deaths (4), in which ischaemic stroke accounting for 78% of prevalences, 70% of new-onsets, and nearly 40% of deaths, while haemorrhagic stroke accounting for ~30% of new-onsets, but 60% of deaths from stroke (5).

Diabetes, defined as a state of hyperglycaemia, is an important risk factor for CVD; patients with diabetes experience an approximately two-fold risk of CVD onset compared with those without diabetes (6). Evidence also suggested that elevated fasting plasma glucose (FPG) levels, even below the threshold for diabetes, also increased the risk of CVD (7–9), but the extent to which level of postload plasma glucose (PPG) was associated with IHD or stroke remained unresolved. Also, uncertainty persists in the consistency of different glycemia measures in prediction of CVD. Furthermore, potential effect modification of other modifiable risk factors (e.g., obesity, lipids or blood pressure et.al) or demographic features on the relationship between hyperglycaemia and CVD is inconclusive.

A recent study reported that consumption of diets with a high glycemic index or load is relative higher in China than that in other countries (10). In addition, a marked geographical variation in the distributions of both diabetes and cardiovascular complications in China has been documented, in which higher incidences but limited health-care services in Southwest provinces and rural regions were observed (11–13). However, data regarding the association between the modifiable risk factors and CVD in Southwest Chinese population are scarce.

All above indicates that more effective targeted intervention strategies are needed to mitigate the burden of CVD in specific regions with higher risk. Guizhou Province is a multi-ethnic area in Southwest China, with complex topography, relative backward in economy and cultural environment. In this study, we aimed to provide an insight to the relationship of glycemia with cardiovascular onsets, by using data from a large cohort study in Guizhou Province, Southwest China.

## Methods

### Study Design and Study Population

The Guizhou Population Health Cohort Study (GPHCS) is a large epidemiological prospective studies in Southwest China, which was established during November 20, 2010 to December 19, 2012. By the multistage proportional stratified cluster sampling method, considering population size, population stability, and local capacity to execute the study protocol and population representative, a total of 9,280 local residents (aged 18 years or above, living in the study regions for more than 6 months and having no plan to move out) from 12 areas (five urban districts and seven rural counties) in Guizhou Province were enrolled into the cohort. All participants had completed a questionnaire, joined the physical examination and signed a written informed consent form at baseline. Ethic approval was obtained from the ethic review board of Guizhou Province (No.S2017-02).

**Figure 1:**
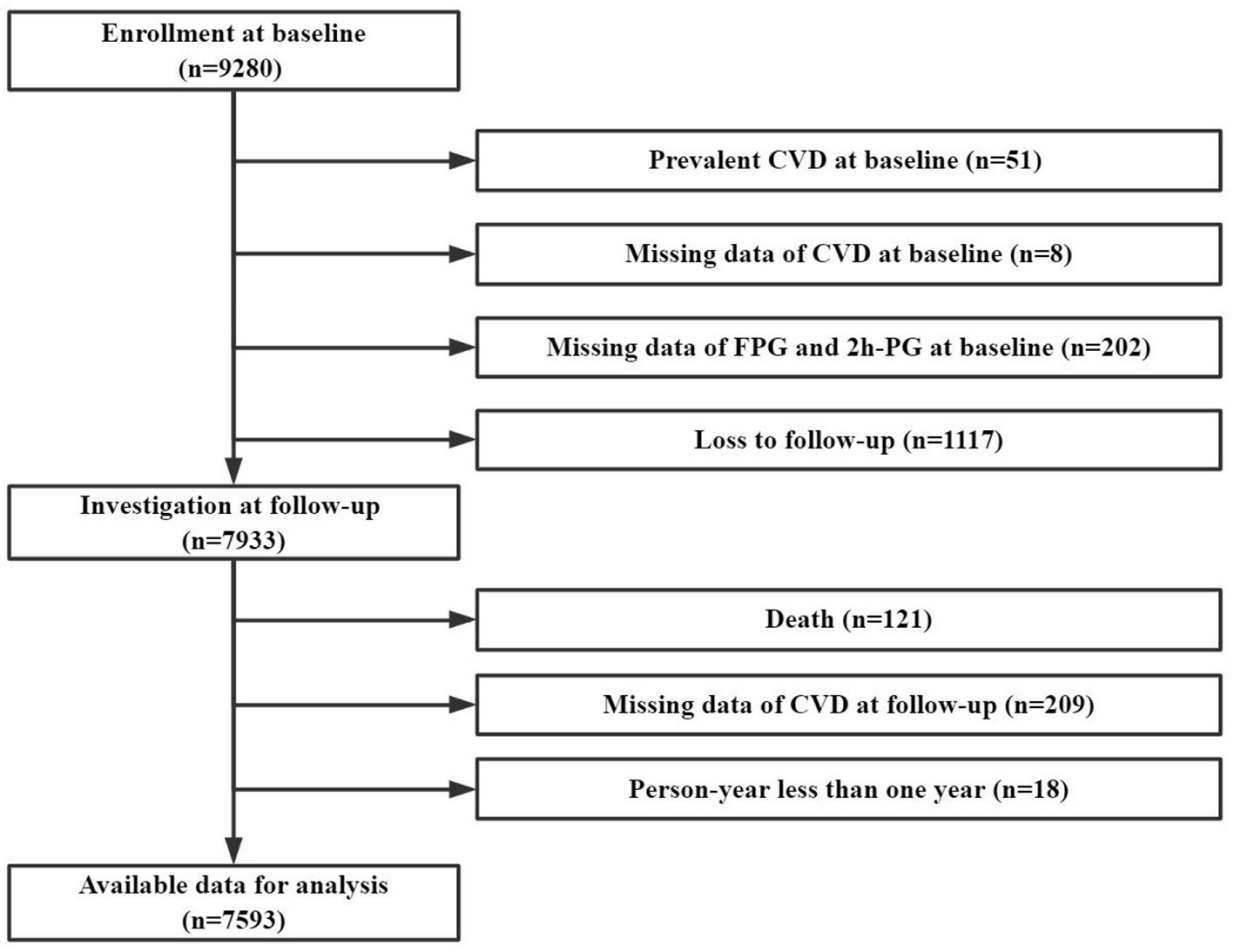
Flow chart.

After excluding those with CVD at baseline, loss to follow-up, death, and person year <1 year, the current study included 7,593 participants with complete data of baseline glycemia and CVD outcome (Figure 1).

### Outcomes of Interest

The primary outcomes were the first onset of a composite of or one of major cardiovascular events, including ischaemic stroke, haemorrhagic stroke and myocardial infarction, which coded by the International Classification of Diseases 10th revision (ICD-10): ischemic stroke (I63), haemorrhagic stroke (I60-61) and myocardial infarction (I21). All reported events were reviewed and integrated centrally by trained clinical physicians. Each participant was followed up until the first occurrence of the corresponding outcome, death, or loss to follow-up, which occurred first prior to December 31, 2020. Incidence rate was calculated as the number of incident cases divided by follow-up person-years.

### Data Collections

Standardized questionnaires were used at baseline to collect information on demographic factors (age, gender, ethnic group, area, education level, marriage status, and occupation type), lifestyle factors (smoking, alcohol drinking, tea drinking, and physical activity), and medical history (diabetes, dyslipidemia, and hypertension). Smoking was defined as smoking at least one cigarette per day for 12 months or more. Alcohol drinking was defined as drinking at least three times per week for 12 months or more. The physical activity was firstly assessed by the duration and frequency of each activity, then weighted by the estimates of the metabolic equivalent (MET) of each activity and summed for all activities performed. The average MET hours per day were used to present the overall physical activity level for each participants.

Anthropometric measurements were taken by trained technicians, including height, weight, waist circumference, and blood pressure, using calibrated instruments with standard protocols. Height, weight, waist circumference were recorded once and usually to the nearest 0.1 cm or 0.1 kg. Waist-to-height ratio (WHtR) was calculated as WC in centimeters divided by height in meters: WHtR = WC÷H; BMI was calculated as weight in kilograms divided by the square of height in meters: BMI (kg/m^2^) = W÷(H÷100)^2^ (H: height, cm; W: weight, kg; WC: waist circumference, cm). Blood pressure was measured three times with a 3-min interval from the left arm after the participant rest in a seated position, and the recorded values of systolic blood pressure (SBP) and diastolic blood pressure (DBP) were calculated as the mean of the last two of three consecutive measurements.

All participants provided a 10-ml blood sample after an overnight fast at least 10 h. They also received an oral glucose tolerance test (OGTT), and the plasma was obtained at 2 h during the test. Concentrations of fasting plasma glucose (FPG) and 2-h postload glucose (2h-PG) were analyzed locally by using the glucose oxidase methods (Roche Diagnostics, Mannheim, Germany). Serum triglycerides (TG), total cholesterol (CHOL), low density lipoprotein cholesterol (LDL-C), and high density lipoprotein cholesterol (HDL-C) in the fasting blood were measured using enzymatic methods (Roche Diagnostics, Mannheim, Germany).

### Diagnostic Criteria

Normal range of FPG and 2h-PG was defined as the value below the threshold of 6.1 mmol/L and 7.8 mmol/L, respectively. Diabetes was defined as those above the threshold of glycemia (FPG≥6.1 mmol/L or 2h-PG≥7.8 mmol/L), having a reported diabetes history, or experiencing anti-diabetes medications (14). Hypertension was defined as abnormal level of current blood pressure (SBP>140 mmHg or DBP>90 mmHg), having a reported hypertension history, or experiencing anti-hypertension medications (14). Dyslipidemia was defined as abnormal level of current blood lipids (TG ≥ 1.7 mmol/L, CHOL ≥ 5.2 mmol/L, LDL ≥ 3.4 mmol/L, HDL < 1.0 mmol/L), having a reported dyslipidemia history, or experiencing anti-dyslipidemia medications (14). General overweight or obesity was defined as BMI ≥ 24 kg/m^2^, central obesity was defined as WC ≥ 85 cm for female or ≥ 90 cm for male, and obesity status was defined as having either of these two types of obesity (15).

Participants with different level of SBP, TG and BMI were divided into two subgroups according to the corresponding cut-off values (14). Participants in the *Normal* group satisfied one of the following conditions: SBP < 120 mm/Hg, TG < 1.7 mmol/L, BMI < 24 kg/m^2^; others were in the *Abnormal* group: SBP ≥ 120 mm/Hg, TG ≥ 1.7 mmol/, BMI ≥ 24 kg/m^2^.

### Statistical Analysis

Continuous variables were presented as means and standard deviations (mean±SD) and compared by using Student's *t*-test. Categorical variables were expressed as frequencies and percentages (*n*, %) and compared by using Chi-square test.

**Figure 2:**
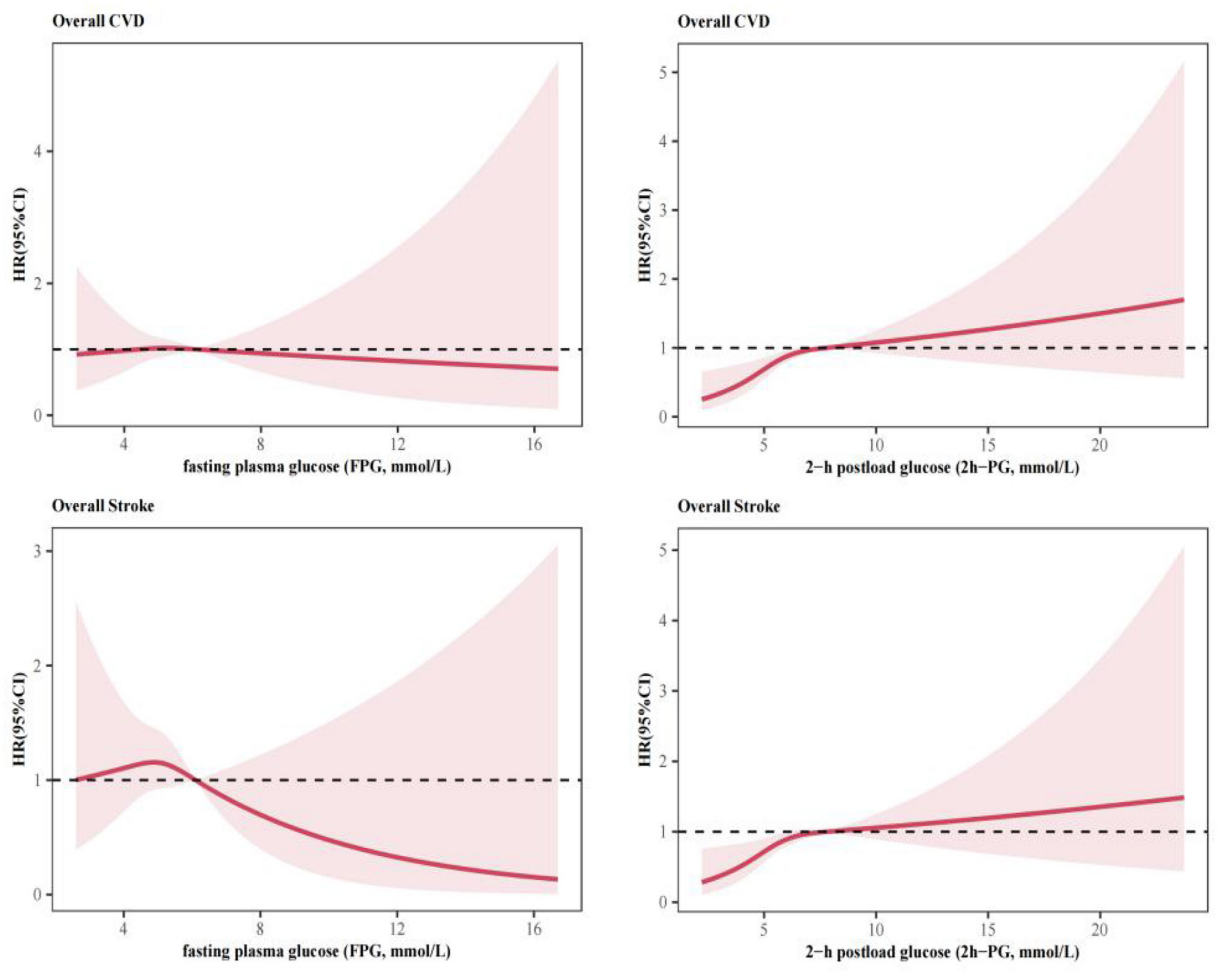
Association of baseline fasting plasma glucose (FPG) or 2-h postload glucose (2h-PG) with cardiovascular diseases (CVD) by using restricted cubic-spline (RCS) plots with three knots.

Restricted cubic-spline (RCS) plots with three knots were used to explore the shape of the association of FPG or 2h-PG with the primary outcomes (Figure 2). Age-adjusted and multi-adjusted cox proportional hazards models were used to estimate hazard ratios (HRs) and 95% confidence intervals (95%CIs) for each tertile of or per one SD increase in baseline level of FPG and 2h-PG. Analyses were also conducted among participants within normal glycemia level and free of diabetes. Considering the possible effect modification by metabolic states, we repeated the above analysis in participants stratified by baseline level of SBP, TG or BMI, as well as the status of hypertension, dyslipidemia or obesity. Subgroup analysis were also performed in subgroups stratified by demographic factors (age, gender, ethnic group, and area).

The level of statistical significance was defined as α = 0.05 of two-side probability. All analyses and figures were performed by using R program (version 4.1.0, R Foundation for Statistical Computing, Vienna, Austria).

## Results

### Descriptive Analysis

Overall, the mean age of 7,593 participants at entry was 43.97 ± 14.92 years, of which 3,609 (47.5%) were male, 4,469 (58.9%) were ethnic Han, and 5,093 (67.1%) resided in rural counties. The mean baseline level of FPG and 2h-PG was 5.21 ± 1.16 mmol/L and 5.88 ± 2.16 mmol/L, respectively. FPG was strongly associated with 2h-PG (r = 0.57, *P* < 0.001).

During a median follow-up of 6.58 years (range 1.10–9.53 years), 174 participants experienced at least one CVD events, including 158 stroke (126 ischemic stroke and 39 Ischemic stroke events), and 24 myocardial infarction. The distributions of baseline characteristics were broadly similar across those with different CVD (Table 1).

**Table 1:** Baseline characteristics of participants according to major cardiovascular disease (CVD) onset.

**Characteristics**	**Total (*n* = 7593)**	**Non-CVD** **(*n* = 7419)**	**CVD (*****n*** **=** **174)**				
			**Overall CVD (*n* = 174)^**b**^**	**Overall Stroke** **(*n* = 158)^**b**^**	**Ischemic stroke (*n* = 126)^**b**^**	**Haemorrhagic stroke** **(*n* = 39)^**b**^**	**Myocardial infarction (*n* = 24)^**b**^**
**Demographic factors**							
Age (years, mean ±SD)	43.97 ± 14.92	43.75 ± 14.87	53.44 ± 13.85^***^	53.55 ± 13.84^***^	54.27 ± 14.02^***^	53.52 ± 14.23^***^	52.54 ± 15.18^***^
Male (*n*,%)	3609 (47.5)	3522 (47.5)	87 (50.0)	78 (49.4)	58 (46.0)	22 (56.4)	13 (54.2)
Ethnic Han (*n*,%)	4469 (58.9)	4348 (58.6)	121 (69.5)^**^	108 (68.4)^*^	91 (72.2)^**^	19 (48.7)	18 (75.0)^**^
Rural (*n*,%)	5093 (67.1)	4966 (66.9)	127 (73.0)	116 (73.4)	92 (73.0)	30 (76.9)	17 (70.8)
Junior middle school and below (*n*,%)	6608 (87.0)	6456 (87.0)	152 (87.3)^*^	137 (86.7)	109 (86.5)^*^	34 (87.2)	21 (87.5)
Married/Cohabit (*n*,%)	6157 (81.1)	6018 (81.1)	139 (79.9)	126 (79.7)	99 (78.6)	32 (82.1)	19 (79.2)
Farmers (*n*,%)	4378 (57.7)	4271 (57.6)	107 (61.5)	97 (61.4)	76 (60.3)	25 (64.1)	16 (66.7)
**Lifestyle factors**							
Smoking (*n*,%)	1941 (25.6)	1890 (25.5)	51 (29.3)	48 (30.4)	39 (31.0)	9 (23.1)	5 (20.8)
Alcohol drinking (*n*,%)	1750 (23.0)	1716 (23.1)	34 (19.5)	30 (19.0)	24 (19.0)	6 (15.4)	5 (20.8)
Tea drinking (*n*,%)^a^	2739 (36.1)	2664 (35.9)	75 (43.1)	69 (43.7)	54 (42.9)	15 (38.5)	11 (45.8)
MET (per day, *n*,%)	109.96 ± 122.45	109.92 ± 122.57	111.75 ± 117.64	110.31 ± 116.10	105.83 ± 118.45	115.16 ± 103.31	121.49 ± 147.67
**Medical history**							
Diabetes (*n*, %)	544 (7.2)	527 (7.1)	17 (9.8)	15 (9.5)	11 (8.7)	4 (10.3)	4 (16.7)
Dyslipidemia (*n*, %)	4300 (56.6)	4199 (56.6)	101 (58.0)	92 (58.2)	72 (57.1)	24 (61.5)	15 (62.5)
Hypertension (*n*, %)	1909 (25.1)	1837 (24.8)	72 (41.4)^***^	66 (41.8)^***^	53 (42.1)^***^	17 (43.6)^***^	10 (41.7)^***^
**Anthropometry and** **metabolic indices**							
BMI (kg/m^2^)^a^	22.86 ± 3.34	22.86 ± 3.35	22.99 ± 3.11	22.99 ± 3.04	23.06 ± 3.12	22.67 ± 2.78	22.87 ± 3.96
WC (cm, mean ± SD)^a^	76.51 ± 9.43	76.48 ± 9.43	77.87 ± 9.66^*^	78.07 ± 9.49^*^	78.37 ± 9.68^*^	76.97 ± 9.04	75.63 ± 12.67
WHtR (mean ± SD)^a^	0.49 ± 0.06	0.49 ± 0.06	0.50 ± 0.07	0.50 ± 0.07	0.50 ± 0.07	0.49 ± 0.06	0.48 ± 0.08
FPG (mmol/L, mean ± SD)	5.21 ± 1.16	5.21 ± 1.16	5.22 ± 1.16	5.13 ± 0.92	5.15 ± 0.85	5.14 ± 1.11	5.86 ± 2.01^**^
2h-PG (mmol/L, mean SD)	5.88 ± 2.16	5.87 ± 2.15	6.41 ± 2.32^**^	6.33 ± 2.22^**^	6.39 ± 2.30^**^	6.15 ± 1.83^**^	7.08 ± 2.91^**^
TG (mmol/L, mean ± SD)^a^	1.73 ± 1.53	1.73 ± 1.53	1.78 ± 1.56	1.76 ± 1.58	1.67 ± 1.11	2.11 ± 2.51	1.89 ± 1.26
CHOL (mmol/L, mean ± SD)^a^	4.78 ± 1.31	4.78 ± 1.29	4.93 ± 1.78	4.88 ± 1.83	4.77 ± 1.57	5.33 ± 2.52^**^	5.08 ± 1.64
HDL-C (mmol/L, mean ± SD)^a^	1.45 ± 0.55	1.45 ± 0.55	1.43 ± 0.61	1.43 ± 0.62	1.44 ± 0.65	1.43 ± 0.46	1.34 ± 0.53
LDL-C (mmol/L, mean ± SD)^a^	2.66 ± 1.17	2.66 ± 1.16	2.57 ± 1.35	2.53 ± 1.35	2.49 ± 1.29	2.79 ± 1.64	2.65 ± 1.39
SBP (mmHg, mean ± SD)^a^	124.87 ± 20.76	124.67 ± 20.63	133.28 ± 24.26^***^	133.27 ± 24.04^***^	133.54 ± 24.46^***^	135.77 ± 26.20^***^	132.72 ± 24.42^***^
DBP (mmHg, mean ± SD)^a^	78.17 ± 11.86	78.08 ± 11.80	82.27 ± 13.81^***^	81.90 ± 13.61^***^	81.93 ± 13.36^***^	82.36 ± 15.89^***^	83.55 ± 15.05^***^

*CVD, cardiovascular diseases; SD, standard deviation; MET, metabolic equivalent; BMI, body mass index; Waist circumference, WC; WHtR, waist-to-height ratio; FPG, fasting plasma glucose; 2h-PG, 2-h postload glucose;TG, triglyceride; CHOL, total cholesterol; HDL-C, high density lipoprotein cholesterol; LDL-C, low density lipoprotein cholesterol; SBP, systolic blood pressure; DBP, diastolic blood pressure*.

a *With missing value*.

b *Compared with those without CVD onset:*

* *P < 0.05;*

** *P < 0.01;*

*** *P < 0.001*.

### Pooled Analysis

In the spline regression analysis, there seemed to be linear associations of overall CVD with FPG and 2h-PG (Figure 2). The risk of CVD events significantly elevated with increasing 2h-PG (Table 2). Participants in the highest tertile of 2h-PG had a 1.87-fold (95%CI: 1.26–2.77) increased risk for overall CVD and a 1.82-fold (95%CI: 1.20–2.75) increased risk for overall Stroke, respectively, compared with those in the lowest tertile, after adjustment for age, sex, smoking, ethnic group, education level, SBP, TG, BMI and WC in Model 2. Similar associations were observed with ischemic stroke, with the relevant multi-adjusted HR of 1.66 (95%CI: 1.04–2.66, Tertile 3 vs Tertile 1, Model 2). No association of 2h-PG with haemorrhagic stroke or myocardial infarction was observed. FPG, if evaluated in tertile category, was unrelated to major CVD events. However, per 1 SD increase in FPG was associated with risk for myocardial infarction, with the multi-adjusted HR of 1.34 (95%CI: 1.07–1.69, Model 2).

**Table 2:** Hazard ratios (HRs) and 95% confidence intervals (95%CIs) for cardiovascular disease (CVD) associated with baseline fasting plasma glucose (FPG) and 2-h postload glucose (2h-PG) according to cox proportional hazards regression models.

	**Fasting plasma glucose (FPG, mmol/L)** ^**a**^				**2-h postload glucose (2h-PG, mmol/L)** ^**b**^			
	**Tertile**			**Per 1-SD** **increase**	**Tertile**			**Per 1-SD** **increase**
	**Tertile 1** **(*n* = 2515)**	**Tertile 2** **(*n* = 2523)**	**Tertile 3** **(*n* = 2555)**		**Tertile 1** **(*n* = 2530)**	**Tertile 2** **(*n* = 2518)**	**Tertile 3** **(*n* = 2545)**	
**Overall CVD**								
Number of cases	51	64	59	-	37	63	74	-
Person-years	17745.57	17758.06	18420.84	-	17816.59	17835.26	18272.62	-
Incident density(cases per 1000 Person-years)	2.87	3.60	3.20	-	2.08	3.53	4.05	-
Model 1^c^	1.00	1.24 (0.86–1.79)	1.05 (0.72–1.52)	0.98 (0.84–1.15)	1.00	1.68 (1.12–2.52)^*^	1.87 (1.26–2.77)^**^	1.17 (1.06–1.31)^**^
Model 2^c^	1.00	1.20 (0.81–1.77)	0.98 (0.65–1.45)	0.96 (0.82–1.14)	1.00	1.69 (1.08–2.64)^*^	1.87 (1.22–2.89)^**^	1.15 (1.02–1.29)^*^
Model 3^c^	1.00	1.17 (0.81–1.69)	1.02 (0.70–1.49)	0.97 (0.83–1.14)	1.00	1.65 (1.10–2.49)^*^	1.74 (1.17–2.60)^**^	1.15 (1.03–1.28)^*^
**Overall Stroke**								
Number of cases	49	57	52	-	34	58	66	-
Person-years	17745.76	17773.75	18432.65	-	17818.88	17844.9	18288.39	-
Incident density (cases per 1000 Person-years)	2.76	3.21	2.82	-	1.91	3.25	3.61	-
Model 1^c^	1.00	1.15 (0.78–1.68)	0.96 (0.65–1.42)	0.90 (0.74–1.08)	1.00	1.68 (1.10–2.57)^*^	1.82 (1.20–2.75)^**^	1.15 (1.03–1.29)^*^
Model 2^c^	1.00	1.13 (0.75–1.70)	0.92 (0.61–1.40)	0.87 (0.71–1.06)	1.00	1.69 (1.06–2.68)^*^	1.81 (1.15–2.84)^*^	1.12 (0.99–1.28)
Model 3^c^	1.00	1.09 (0.74–1.59)	0.94 (0.63–1.39)	0.88 (0.73–1.07)	1.00	1.66 (1.09–2.54)^*^	1.70 (1.12–2.59)^*^	1.13 (1.00–1.27)
**Ischemic stroke**								
Number of cases	40	46	40	-	26	48	52	-
Person-years	17835.74	17868.71	18515.04	-	17880.12	17924.11	18415.26	-
Incident density (cases per 1000 Person-years)	2.24	2.57	2.16	-	1.45	2.68	2.82	-
Model 1^c^	1.00	1.09 (0.72–1.67)	0.79 (0.51–1.22)	0.87 (0.70–1.07)	1.00	1.69 (1.05–2.73)^*^	1.66 (1.04–2.66)^*^	1.15 (1.01–1.31)^*^
Model 2^c^	1.00	1.07 (0.67–1.69)	0.78 (0.49–1.25)	0.86 (0.68–1.08)	1.00	1.79 (1.05–3.06)^*^	1.75 (1.04–2.97)	1.13 (0.98–1.29)
Model 3^c^	1.00	1.00 (0.66–1.53)	0.78 (0.50–1.21)	0.86 (0.69–1.07)	1.00	1.65 (1.02–2.67)^*^	1.53 (0.95–2.46)	1.12 (0.98–1.28)
**Haemorrhagic stroke**								
Number of cases	10	13	16	-	9	12	18	-
Person-years	17817.08	17855.85	18497.89	-	17864.54	17924.72	18381.57	-
Incident density (cases per 1000 Person-years)	0.56	0.73	0.86	-	0.50	0.67	0.98	-
Model 1^c^	1.00	1.29 (0.56–2.94)	1.46 (0.66–3.22)	0.91 (0.63–1.31)	1.00	1.33 (0.56–3.16)	1.88 (0.84–4.18)	1.10 (0.84–1.43)
Model 2^c^	1.00	1.22 (0.53–2.83)	1.23 (0.55–2.77)	0.83 (0.56–1.24)	1.00	1.22 (0.50–2.95)	1.66 (0.73–3.76)	1.01 (0.74–1.38)
Model 3^c^	1.00	1.28 (0.56–2.94)	1.36 (0.61–3.01)	0.88 (0.61–1.27)	1.00	1.38 (0.58–3.29)	1.87 (0.83–4.19)	1.08 (0.82–1.41)
**Myocardial infarction**								
Number of cases	4	10	10	-	4	7	13	-
Person-years	17841.37	17868.71	18522.23	-	17885.75	17931.3	18415.26	-
Incident density (cases per 1000 Person-years)	0.22	0.56	0.54	-	0.22	0.39	0.71	-
Model 1^c^	1.00	2.44 (0.77–7.79)	2.14 (0.67–6.83)	1.27 (1.06–1.54)^*^	1.00	1.72 (0.50–5.88)	2.89 (0.94–8.88)	1.31 (1.06–1.63)^*^
Model 2^c^	1.00	2.51 (0.66–9.48)	2.29 (0.61–8.58)	1.28 (1.05–1.54)^*^	1.00	1.53 (0.37–6.44)	3.05 (0.85–11.00)	1.34 (1.07–1.69)^*^
Model 3^c^	1.00	2.28 (0.71–7.28)	2.07 (0.64–6.67)	1.28 (1.05–1.56)^*^	1.00	1.72 (0.50–5.92)	2.71 (0.87–8.45)	1.31 (1.04–1.64)^*^

a *FPG level: Tertile 1, <4.78 mmol/L; Tertile 2, 4.78–5.41 mmol/L; Tertile 3, ≥5.42 mmol/L; 2h-PG level: Tertile 1, <4.99 mmol/L; Tertile 2, 5.00–5.98 mmol/L; Tertile 3, ≥5.99 mmol/L*.

c *Model 1: Adjusted for age*.

*Model 2: Model 1 + additionally adjusted for sex, smoking, ethnic group, education level, systolic blood pressure (SBP), triglyceride (TG), body mass index (BMI) and waist circumference (WC)*.

*Model 3: Model 1 + additionally adjusted for sex, smoking, ethnic group, education level, hypertension history, dyslipidemia history, and obesity status*.

* *P < 0.05;*

** *P < 0.01*.

The effect of 2h-PG on CVD risk persisted among those within normal range of FPG (Table 3). The corresponding multi-adjusted HRs were 1.81(95%CI: 1.17–2.82, Model 2) for overall CVD, 1.70(95%CI: 1.12–2.59, Model 2) for overall Stroke, and 1.64(95%CI: 1.03–2.62, Model 2) for ischaemic stroke, respectively, comparing those in the highest tertile of 2h-PG with those in the lowest tertile. Similar results were observed among participants with normal 2h-PG and free of diabetes. Additionally, per 1 SD increase in 2h-PG added the risk for myocardial infarction by 31% (HR:1.31, 95%CI: 1.04-1.64, Model 2). By contrary, FPG still showed no association with CVD risk among those subgroups, which was consistent with the results in the pooled analysis.

**Table 3:** Hazard ratios (HRs) and 95% confidence intervals (95%CIs) for cardiovascular disease (CVD) associated with baseline fasting plasma glucose (FPG) and 2-h postload glucose (2h-PG) among participants within normal baseline blood glucose level or free of diabetes mellitus (DM).

	**Fasting plasma glucose (FPG, mmol/L)** ^**a**^				**2-h postload glucose (2h-PG, mmol/L)** ^**b**^			
	**Tertile**			**Per 1-SD** **increase**	**Tertile**			**Per 1-SD** **increase**
	**Tertile 1**	**Tertile 2**	**Tertile 3**		**Tertile 1**	**Tertile 2**	**Tertile 3**	
**Participants within normal FPG level (*****n*** **=** **6683)**^**c**^								
Overall CVD	1.00	1.13 (0.76–1.68)	1.17 (0.79–1.73)	1.01 (0.86–1.19)	1.00	1.68 (1.08–2.60)^*^	1.85 (1.21–2.83)^**^	1.20 (1.06–1.34)^**^
Overall Stroke	1.00	1.06 (0.70–1.60)	1.12 (0.75–1.68)	0.99 (0.84–1.16)	1.00	1.63 (1.04–2.57)^*^	1.81 (1.17–2.82)^**^	1.20 (1.06–1.35)^**^
Ischemic stroke	1.00	1.06 (0.67–1.68)	1.03 (0.65–1.62)	0.99 (0.82–1.19)	1.00	1.78 (1.06–2.98)^*^	1.75 (1.05–2.90)^*^	1.19 (1.05–1.36)^**^
Haemorrhagic stroke	1.00	0.98 (0.41–2.35)	1.16 (0.51–2.65)	0.89 (0.65–1.23)	1.00	0.91 (0.35–2.38)	1.67 (0.73–3.81)	1.04 (0.77–1.41)
Myocardial infarction	1.00	1.72 (0.52–5.76)	1.74 (0.52–5.82)	1.44 (0.88–2.36)	1.00	1.95 (0.48–7.88)	3.06 (0.84–11.10)	1.29 (0.97–1.72)
**Participants within normal 2h-PG level (*****n*** **=** **6838)**^**c**^								
Overall CVD	1.00	1.17 (0.81–1.69)	1.02 (0.70–1.49)	0.97 (0.83–1.14)	1.00	1.65 (1.10–2.49)^*^	1.74 (1.17–2.60)^**^	1.15 (1.03–1.28)^*^
Overall Stroke	1.00	1.09 (0.74–1.59)	0.94 (0.63–1.39)	0.88 (0.73–1.07)	1.00	1.66 (1.09–2.54)^*^	1.70 (1.12–2.59)^*^	1.13 (1.00–1.27)
Ischemic stroke	1.00	1.00 (0.66–1.53)	0.78 (0.50–1.21)	0.86 (0.69–1.07)	1.00	1.65 (1.02–2.67)^*^	1.53 (0.95–2.46)	1.12 (0.98–1.28)
Haemorrhagic stroke	1.00	1.28 (0.56–2.94)	1.36 (0.61–3.01)	0.88 (0.61–1.27)	1.00	1.38 (0.58–3.29)	1.87 (0.83–4.19)	1.08 (0.82–1.41)
Myocardial infarction	1.00	2.28 (0.71–7.28)	2.07 (0.64–6.67)	1.28 (1.05–1.56)^*^	1.00	1.72 (0.50–5.92)	2.71 (0.87–8.45)	1.31 (1.04–1.64)^*^
**Participants without diabetes mellitus (DM) (*****n*** **=** **6203)**^**c**^								
Overall CVD	1.00	1.09 (0.72–1.67)	1.17 (0.77–1.76)	0.99 (0.84–1.17)	1.00	1.82 (1.15–2.87)^*^	1.76 (1.12–2.77)^*^	1.22 (1.04–1.44)^*^
Overall Stroke	1.00	1.06 (0.68–1.64)	1.09 (0.71–1.67)	0.96 (0.80–1.14)	1.00	1.70 (1.06–2.72)^*^	1.64 (1.03–2.62)^*^	1.21 (1.02–1.44)^*^
Ischemic stroke	1.00	1.06 (0.65–1.70)	0.96 (0.59–1.55)	0.95 (0.78–1.14)	1.00	1.99 (1.17–3.40)^*^	1.69 (0.99–2.90)	1.21 (1.00–1.46)
Haemorrhagic stroke	1.00	1.00 (0.37–2.66)	1.23 (0.49–3.06)	0.90 (0.63–1.28)	1.00	0.71 (0.25–2.02)	1.34 (0.56–3.22)	1.07 (0.74–1.55)
Myocardial infarction	1.00	1.12 (0.30–4.20)	1.55 (0.45–5.32)	1.41 (0.81–2.45)	1.00	3.18 (0.64–15.9)	3.70 (0.77–17.7)	1.29 (0.80–2.08)

a *FPG level: (1) for participants within normal FPG level: Tertile 1, <4.70mmol/L; Tertile 2, 4.71–5.24 mmol/L; Tertile 3, ≥5.25 mmol/L; (2) for participants within normal 2PBG level: Tertile 1, <4.78mmol/L; Tertile 2, 4.79–5.40 mmol/L; Tertile 3, ≥5.41 mmol/L; (3) for participants without diabetes mellitus (DM): Tertile 1, <4.68mmol/L; Tertile 2, 4.69–5.21 mmol/L; Tertile 3, ≥5.22 mmol/L*.

b *2h-PG level: (1) for participants within normal FPG level: Tertile 1, <4.92 mmol/L; Tertile 2, 4.93–5.81 mmol/L; Tertile 3, ≥5.82 mmol/L; (2) for participants within normal 2PBG level: Tertile 1, <5.00mmol/L; Tertile 2, 5.01–5.98 mmol/L; Tertile 3, ≥5.99 mmol/L; (3) for participants without diabetes mellitus (DM): Tertile 1, <4.87mmol/L; Tertile 2, 4.88–5.69 mmol/L; Tertile 3, ≥5.70 mmol/L*.

c *HRs were based on Model 3*.

* *P < 0.05;*

** *P < 0.01*.

### Stratified Analysis

**Figure 3:**
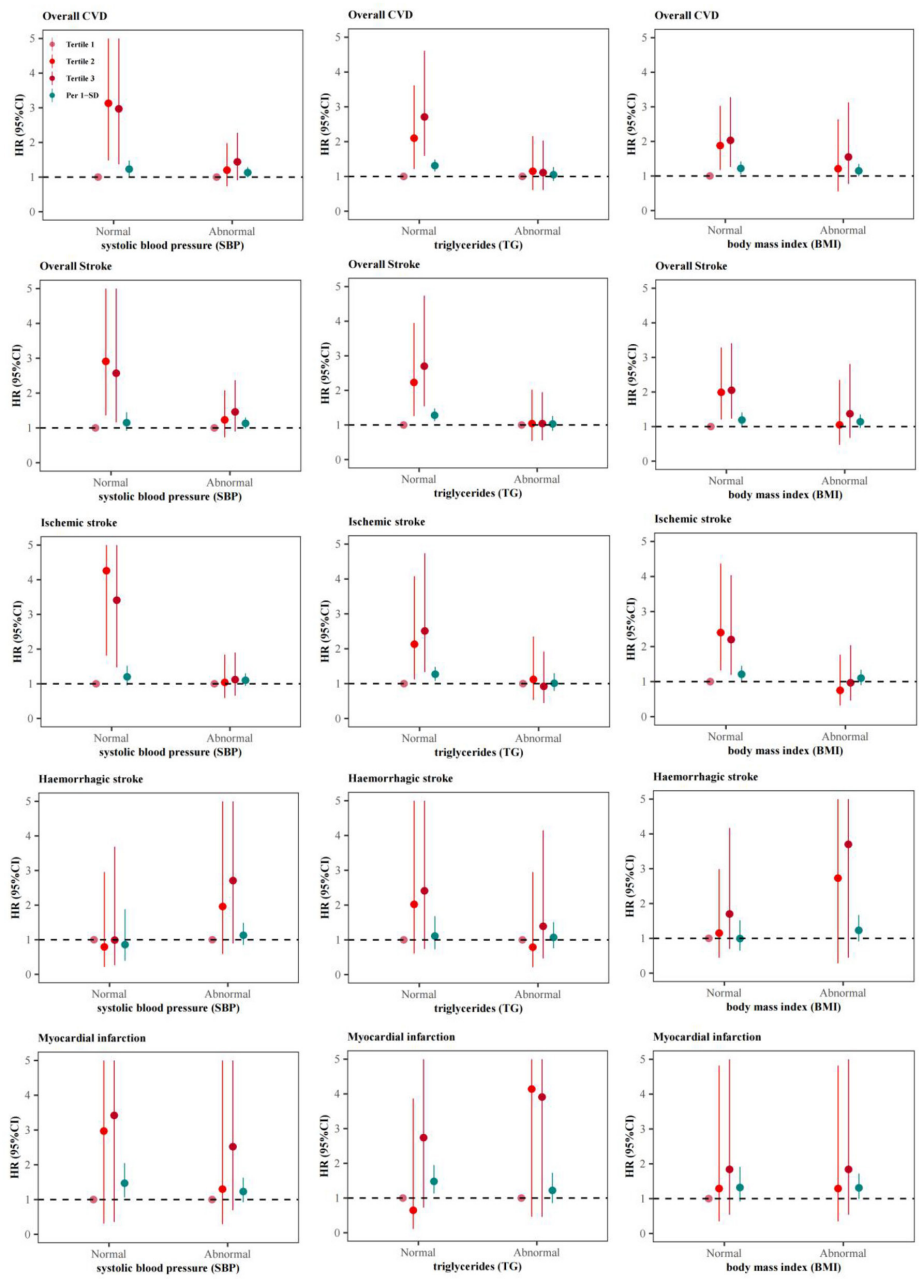
Adjusted hazard ratios (HRs) and 95% confidence intervals (95%CIs) for cardiovascular disease (CVD) associated with baseline 2-h postload glucose (2h-PG) varied by baseline level of SBP, TG or BMI.

**Figure 4:**
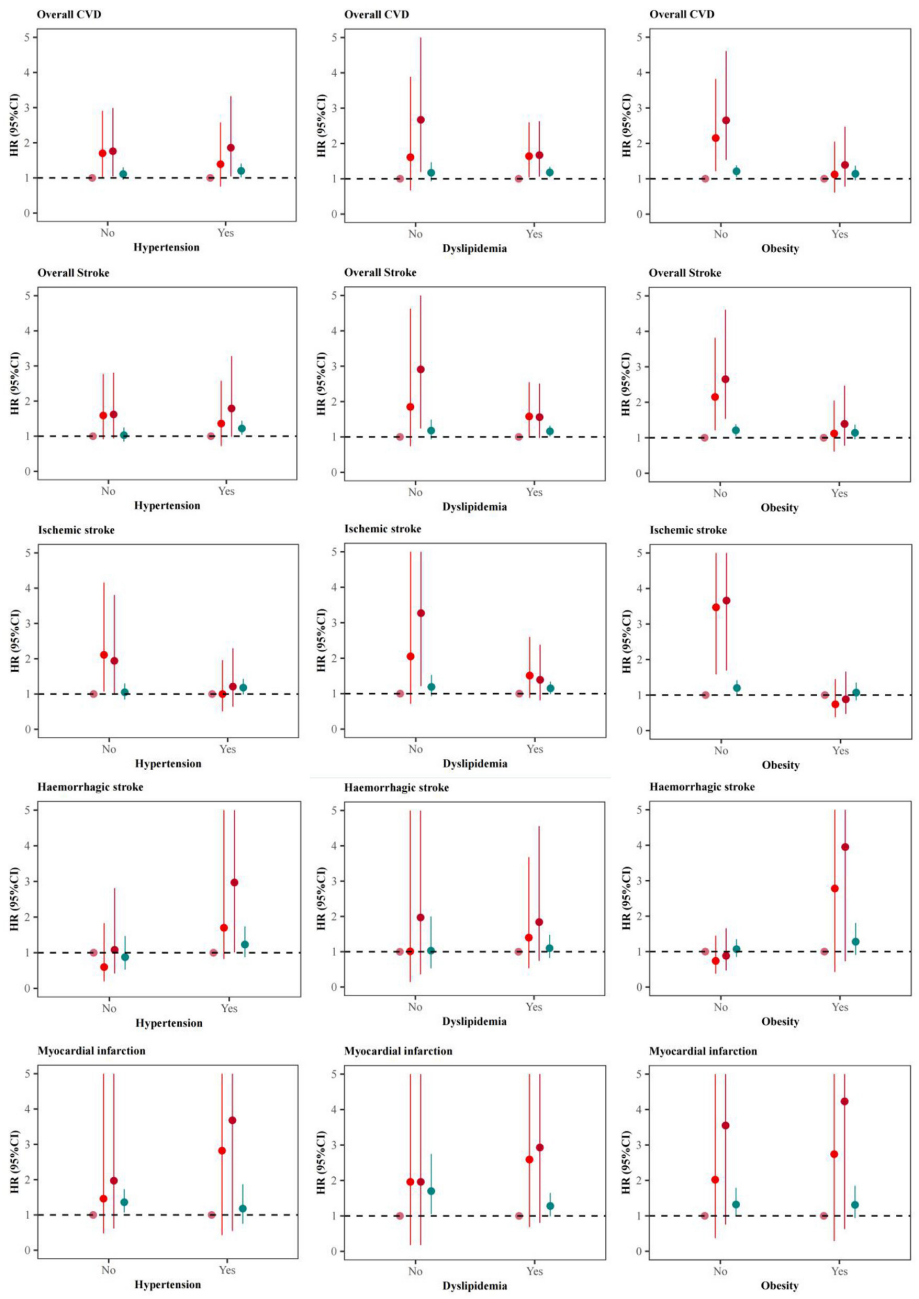
Adjusted hazard ratios (HRs) and 95% confidence intervals (95%CIs) for cardiovascular diseases (CVD) associated with baseline 2-h postload glucose (2h-PG) varied by status of hypertension, dyslipidemia or obesity.

The multi-adjusted HRs for major CVD events predicted by 2h-PG varied across different metabolic risk factors (Figures 3, 4). Among 3,371 (44.40%) with a SBP of <120 mmHg, 4,755 (62.62%) with a TG of <1.7 mmol/L, and 5,189 (68.34%) participants with a BMI of <24kg/m^2^, the risks for major CVD events were more evident than that in pooled analysis (Figure 3), with a maximum multi-adjusted HR of 4.26 (95%CI: 1.81–5.30, Tertile 2 vs Tertile 1, Model 2) for ischemic stroke among those with a normal range of SBP (Figure 3). Similar association trends were shown in those without hypertension, dyslipidemia and obesity (Figure 4). However, the relationship of 2h-PG with CVD events disappeared among those with abnormal level of SBP, TG or BMI, or having hypertension, dyslipidemia or obesity.

**Figure 5:**
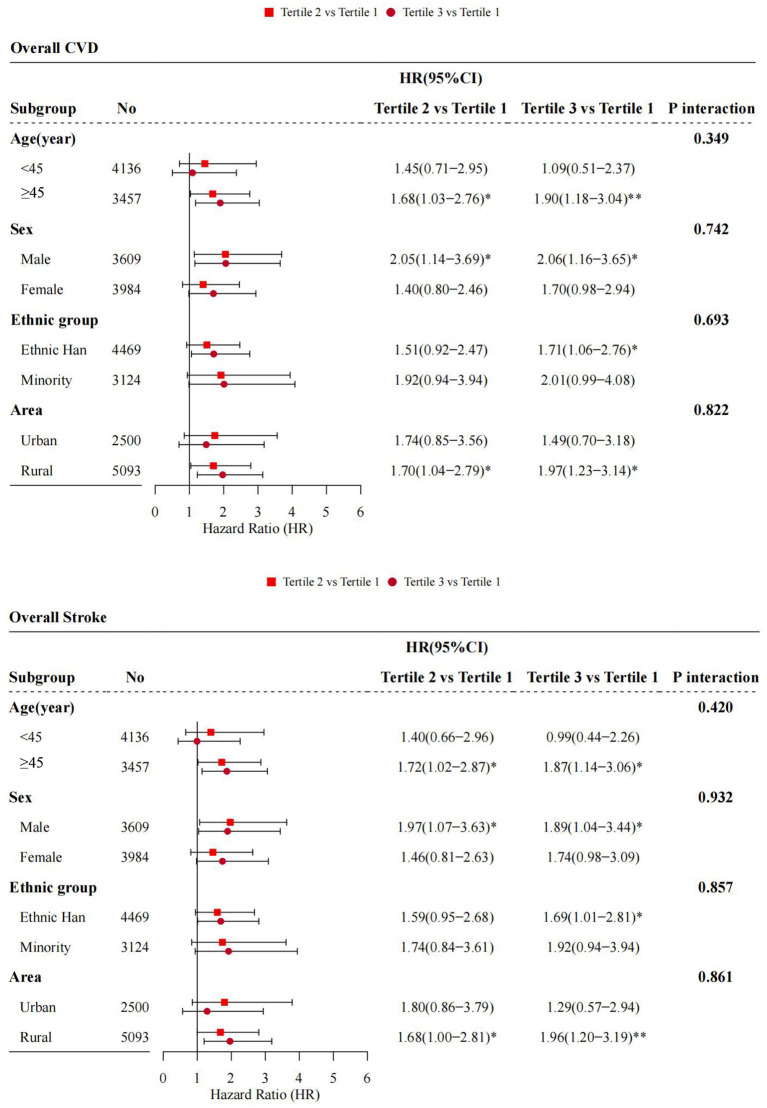
Adjusted hazard ratios (HRs) and 95% confidence intervals (95%CIs) for cardiovascular disease (CVD) associated with baseline 2-h postload glucose (2h-PG) across baseline demographic factors. *P < 0.05; **P < 0.01.

Moreover, no statistically significant effect modification by gender, age, ethnic group and residence area were observed (*P*_*forinteraction*_>0.05), although the strengths of the association seemed stronger in those aged more than 45 years at baseline (HR:1.90, 95%CI: 1.18–3.04, Tertile 3 vs. Tertile 1), males (HR:2.06, 95%CI: 1.16–3.65, Tertile 3 vs. Tertile 1), ethnic Han Chinese (HR:1.71, 95%CI: 1.06–2.76, Tertile 3 vs. Tertile 1), and rural residents (HR:1.97, 95%CI: 1.23–3.14, Tertile 3 vs. Tertile 1) (Figure 5).

## Discussions

In this large cohort study in Southwest China, our analysis suggested that elevated 2h-PG, even within the normal range, adds excess risks for CVD onset, particularly ischemic stroke. The associations varied by other metabolic states and demographic factors. Although FPG and 2h-PG were both indicators for diabetes, despite the apparent relationship between FPG and 2h-PG, FPG was unrelated to CVD onset in our study.

Previous prospective studies have reported that 2h-PG was positively associated with the risk of incidence and prognosis of CVD among participants without previous diabetes, while FPG was of no value in the corresponding predictions (16, 17). Other observational studies also observed that prediabetes (intermediate hyperglycaemia, a high-risk state for diabetes with glycemia higher than normal but lower than diabetes thresholds) and diabetes defined by 2h-PG criteria added more predictive information of future cardiovascular and all-cause deaths (18, 19). Our results were consisted with the above findings.

The current study found the association with higher risk of CVD was inverted J-shaped but not significant for FPG and mild linear for 2h-PG, respectively, by using the restricted cubic spline (RCS) analysis. These findings contrasted with an approximately J-shaped association of all glycemic indices with CVD from previous studies (16, 20), indicating that both hypoglycemia and hyperglycemia were likely to increase the risk of CVD. These discrepancies might be interpreted by the differences in methodology and study population. Unexpectedly, we found no association between 2h-PG and the risk of haemorrhagic stroke and myocardial infarction, which was partly due to the limited number of these events. But the importance of 2h-PG in prediction of myocardial infarction has been raised in a cohort study by George et al. (21).

The burden caused by glycemia were substantially underestimated by the reliance on FPG alone in previous studies (16, 17). FPG is measured to estimate the ability to regulate glycemia in the absence of dietary, and has been widely used in clinical practice because its measurement is economical and convenient. However, FPG alone fails to reflect adequately a diurnal profile and long-term levels of glycemia (22). By contrast, PPG represents plasma glucose excursions after food ingestion, which mainly results from a moderate to severe insulin resistance and from an impaired late-phase insulin secretory response to oral glucose (23). Most individuals usually experience a 8-to 12-hour postprandial state a day, so that PPG accounts for one third to a half of diurnal glycemic variability (24). 2h-PG is a typical measure of PPG at 2 h after OGTT, which approximates the time of peak glucose in those with diabetes (25). Collectively, postload hyperglycemia precedes to fasting hyperglycemia and diabetes, as results of a deficiency in insulin secretion and a decline in β-cell function, leading to consequent suppression of hepatic glucose production and decreases in peripheral glucose uptake (26). Chronic hyperglycaemia contributes to diabetic and cardiovascular complications through two mechanisms, namely excessive protein glycation and activation of oxidative stress. These derangements promote inflammatory response and endothelial dysfunction (27).

Importantly, there seems to be no glycemic threshold for prediction of either micro-vascular or macro-vascular complications, which may be far below the diabetic threshold (28, 29). It is also uncertain that whether prediabetes is pathogenic or merely a prelude to the disease state of diabetes (30). In the current study, 2h-PG was shown to increase CVD risk though within the normal range, and similar results were observed among those within normal range of FPG or free of diabetes. Classification of population as either normal glycemia, prediabetes or diabetes appears to neglect the fact that the risk of cardiovascular complications substantially increases among those with glycemia at the higher end of the normal range (31).

Given that the relation between 2h-PG and CVD risk can be confounded by other modifiable cardiovascular metabolic risk factors, including obesity, blood pressure, and triglyceride levels. The current study also conducted the same association analysis in those with different baseline level of BMI, SBP and TG. But the dose-response effects of 2h-PG with increased CVD risk were only observed in those with normal range of these metabolic factors, indicating that 2h-PG remains independently predictive of CVD outcomes. The heterogeneity of the results across participants with different metabolic status may be due to the inadequate sample size and other confounding factors, such as medication and changes in lifestyles.

The strengths of the current study include that larger sample size among few cohorts in Southwest China, multi-ethnic groups, participants with good representative and compliance, a relatively high rate of follow-up completeness (87.96%), and less missing data in both FPG and 2h-PG of each individual. However, there were also several limitations. First, we measured the FPG and 2h-PG once and did not take the physiological fluctuations in blood glucose into account. Second, the number of some CVD sub-type (such as haemorrhagic stroke and myocardial infarction) was too small to have a reliable assessment of glycemia on these onsets. Further studies are warrant to get more evidence. Third, there was a lack of information on glycosylated hemoglobin (HbA_1c_).

In summary, our study contributes to a new knowledge about the importance of glycemic variables in predicting the risk of future cardiovascular events among Southwest Chinese.

## Conclusions

2h-PG, in contrast to FPG, is an important indicator in predication of CVD in Southwest Chinese. Elevated 2h-PG, though below the threshold for diabetes, independently increased the risk of CVD.

## Data Availability Statement

The datasets for this manuscript will be made available upon request, further inquiries can be directed to the corresponding author Tao Liu, liutaombs@163.com and Na Wang, na.wang@fudan.edu.cn.

## Ethics Statement

The studies involving human participants were reviewed and approved by the ethic review board of Guizhou Province (No. S2017-02). The patients/participants provided their written informed consent to participate in this study.

## Author Contributions

YiyW, JZ, and YWu were involved in data collection and assembly. YinW and NW analyzed and interpreted the data. YinW and LY drafted the manuscript as co-first authors. NW, TL, and CF designed the study and reviewed and revised the manuscript. All authors contributed to the preparation of the final document, read, and approved the final manuscript.

## Funding

This work was supported by Guizhou Province Science and Technology Support Program (Qiankehe [2018]2819).

## Conflict of Interest

The authors declare that the research was conducted in the absence of any commercial or financial relationships that could be construed as a potential conflict of interest.

## Publisher's Note

All claims expressed in this article are solely those of the authors and do not necessarily represent those of their affiliated organizations, or those of the publisher, the editors and the reviewers. Any product that may be evaluated in this article, or claim that may be made by its manufacturer, is not guaranteed or endorsed by the publisher.
